# The History and Capabilities of Herbal Simples

**Published:** 1890-11-01

**Authors:** W. T. Fernie


### The History and Capabilities of Herbal Simples.

By W. T. Fernie, M.D.

### XXII.—COLTSFOOT.

Sweet are the uses of adversity, when it takes the form of bronchial catarrh, and asks to be cured by Coltsfoot rock. The confectioner makes this in sugar-sticks of a pale brown colour, fluted lengthways, and flavoured with some essential oil, as is well known to every schoolboy. But its active principle is got from the herb coltsfoot, or *tussilago farfara*, so called from the Latin words *tussis, ago,*—I drive away a cold, and *farfar*,—the white poplar tree, which has a similar leaf, and which the Greeks named *farfarus.* The Coltsfoot belongs to the composite order of plants, and grows abundantly in places of moist, heavy soil, especially along the sides of our upturned railway banks. The seeds must have lain dormant underneath from primitive times; and the rotting foliage of the plant, retaining its juices, is supposed to have promoted the growth and development of our common earthworm. Some of the older botanists named this plant *filius ante patrem*, because the flowers appear and wither before the leaves are produced. These flowers are conspicuous at the commencement of spring, studding the banks with gay, yellow-headed blossoms, each growing on a stiff scaly stalk. The leaves which follow later on are often smoked as British herbal tobacco, and are mixed for this purpose with the dried leaves and flowers of the eyebright, buckbean, betony, thyme, and lavender, to which some persons add rose leaves and chamomile flowers. All these are rubbed together in a coarse powder with the hands; which powder may be very beneficially employed in asthma by smoking it in the usual way; Coltsfoot should form quite one half of the ingredients. From earliest times the plant has been found helpful for coughs and affections of the chest. Hippocrates advised it with honey for "ulcerations of the lungs." Dioscorides, Pliny, and Galen severally commended the use of its smoke conducted into the mouth through a funnel or reed for giving ease to coughs and laborious breathing. In taste the leaves are harsh, bitter, and mucilaginous. They appear in March, being green above, with an under surface which is white and cottony. Sussex peasants think the white down of the leaves a most valuable medicine. Linnæus says, "Et adhuc hodie plebs in Sueciâ instar tabaci contra tussim fugit"—"Even to-day the Swiss people cure their coughs with coltsfoot employed like tobacco." All parts of the plant contain chemically tannin, with a special bitter principle, and free mucilage, so that it may be considered emollient, demulcent, and slightly tonic. The names coltsfoot and horsehoof—*ungula caballina*—which the plant bears, are derived from the shape of the leaf. It is likewise called asses foot—*pas d'ane*, and cough wort, from its use as a pectoral medicine; also fole's foot and bull's foot. To make a decoction or infusion of the herb for chronic bronchitis a pint of boiling water should be poured on an ounce of the dried leaves and flowers, half-a-tea-cupful of which liquid, when cold, may be taken three or four times a day. Dr. Cullen employed a decoction of the leaves with much benefit in scrofula, where the use of sea-water had failed. Dr. Fuller tells of a girl cured of twelve scrofulous sores by drinking daily for four months as much as she could of a tea made so strong from the leaves as to be sweet and glutinous. A modern medicinal drink is brewed with boiling water from the leaves, together with liquorice root and honey added. When the flowers are fully blown and fall off, the seeds, with their pappus, or "clock," form a beautiful head of white flossy silk, and if this flies away when there is no wind, it is said to be a certain sign of rain. The goldfinch often lines her nest with the soft pappus of the coltsfoot. The silky down of the seedheads is used in the Highlands for stuffing pillows; and the presence of coal is said to be indicated by an abundant growth of the plant. A certain preparation called essence of coltsfoot found great favour with our grandparents for treating their colds. This consisted of balsam of Tolu and Friar's balsam in equal parts, whereto was added double the quantity of rectified spirit of wine. It will be seen that it did not contain a particle of Coltsfoot; and the nostrum could not have been harmless in inflammatory coughs because of the spirit in excess. Dr. Paris says, "And this forsooth is a pectoral for coughs"; "if a patient with a catarrh should recover whilst using such a remedy I should certainly designate it a lucky escape rather than a skilful cure."

Much as the valiant Amazon women of ancient Cappadocia cut off one breast in order the better to draw the long bow, so the coltsfoot flower has nobly sacrificed the honey and pollen of its outer ray florets that it might become more conspicuously attractive to insects, and thus secure cross-fertilisation for the blossom at large. "But, hark! I hear the pancake bell!" as said Poor Richard, in his Almanack, 1684, alluding to pancakes then made with coltsfoot, like tansies, and fried with saged butter! Other quaint remedies for colds were given also in those days. Mrs. Delaney writes in 1758, "Does Mary cough in the night?" "Two or three snails boiled in her barley-water may be of great service to her." Gerarde told concerning coltsfoot, "the fume of the dried leaves burned upon coles effectually helpeth those that fetch their winde thicke; and breaketh without peril the impostumes of the "brest"; also, "the green leaves do heal the hot inflammation called Saint Anthony's fire." One old author has derived the name of the plant from "cult's futter"—"cold's food"; and its appellation, tussilago, from "tussis," a cough, and "laganum," a kind of lozenge. The white wool got from the under side of coltsfoot leaves, when mixed with nitre, was formerly employed as the best tinder. Taken altogether, the coltsfoot has been justly termed "Nature's best herb for the lungs, and her most eminent thoracic." The brilliant yellow colour of its flowers helps to show the antiquity of this herb, which may be certainly referred back to Tertiary times. Then the primitive flora was exclusively yellow; whilst next in progressive order came white, pink, red, and blue flowers. The different colours of white light have different properties. Yellow rays yield brightness; red rays produce heat; and blue rays are most necessary for the growth of a plant. Thus it is that most of our flowers are yellow or white in the cold days of early spring; whilst the summer sun bestows on our gardens a rich abundance of red and blue floral splendour. A quaint adage of old folklore ran as follows:—

> "Green is forsaken, and yellow forsworn,
> Blue is the colour that best may be worn."

